# Tumor suppressive role of microRNA-139-5p in bone marrow mesenchymal stem cells-derived extracellular vesicles in bladder cancer through regulation of the KIF3A/p21 axis

**DOI:** 10.1038/s41419-022-04936-0

**Published:** 2022-07-12

**Authors:** Ying Xiang, Dong Lv, Tao Song, Chao Niu, Ying Wang

**Affiliations:** 1 Department of Urology, Sichuan Academy of Medical Sciences & Sichuan Provincial People’s Hospital, Chengdu, 610072 P. R. China; 2 Department of Urology, Nanchong Central Hospital, The Second Clinical Affiliated College of North Sichuan Medical College, Nanchong, 637000 P. R. China; 3 Department of Oncology, Sichuan Academy of Medical Sciences & Sichuan Provincial People’s Hospital, Chengdu, 610072 P. R. China

**Keywords:** Cancer, Diseases

## Abstract

The emerging roles of extracellular vesicles (EVs) in bladder cancer have recently been identified. This study aims to elucidate the role of microRNA-139-5p (miR-139-5p) shuttled by bone marrow mesenchymal stem cells (BMSCs)-derived EVs (BMSCs-EVs) in bladder cancer, with the possible mechanism explored. Expression of miR-139-5p and KIF3A was tested, followed by an analysis of their correlation. EVs were isolated from BMSCs and co-cultured with T24 or BOY-12E cells with miR-139-5p mimic/inhibitor, oe-KIF3A, and/or si-p21 transfected to study the roles of miR-139-5p/KIF3A/p21 in bladder cancer cell functions. A nude mouse model of subcutaneous xenograft tumor was constructed to detect the effect of miR-139-5p in BMSCs-EVs on the tumorigenesis and lung metastasis of bladder cancer cells in vivo. It was identified that miR-139-5p was highly expressed in BMSCs-EVs, but poorly expressed in bladder cancer. BMSCs-EVs transferred miR-139-5p into bladder cancer cells where miR-139-5p inhibited the malignant features of bladder cancer cells in vitro. miR-139-5p in BMSCs-EVs targeted KIF3A and inhibited the expression of KIF3A, thereby activating p21. miR-139-5p in BMSCs-EVs arrested the tumorigenesis and lung metastasis of bladder cancer cells in vivo by modulation of the KIF3A/p21 axis. Altogether, BMSCs-EVs carried miR-139-5p targeted KIF3A to activate p21, thus delaying the occurrence of bladder cancer.

## Introduction

As one of the most common genitourinary system cancers, bladder cancer causes ~150,000 cases of death every year in the world [1]. Statistics has been noted that the incidence of bladder cancer in men is three to four times that of women [2]. As one complex pathological process, the main treatment modalities of bladder cancer include surgical resection, radiotherapy, chemotherapy as well as comprehensive treatment [3]. At present, the main challenge regarding bladder cancer treatment is the high recurrence rate after drug treatment and resection [4]. Particularly, patients with bladder cancer, especially those with muscle-invasive ones, have a poor prognosis despite receiving systemic treatment [5]. Thus, a fully understanding of its pathological condition and new treatment direction are urgently needed.

Extracellular vesicles (EVs) are natural components secreted by cells, with long circulation, good bio-compatibility, and physiological barrier crossing ability [6]. Bone marrow mesenchymal stem cells (BMSCs)-derived EVs (BMSCs-EVs) have been found to inhibit T24 cell proliferation by blocking the cell cycle of T24 cells, and induce T24 cell apoptosis [7]. Besides, EVs are important mediators for the transfer of microRNAs (miRNAs) [8]. miRNAs are small non-coding RNAs that can control gene expression at multiple levels [9]. As earlier report, miR-139-5p shares a close correlation with bladder cancer development [10]. Also, low miR-139-5p is detected in bladder cancer, while its elevation decreased the proliferation and limits the migratory and invasive features of T24 cells [11]. In addition, kinesin family member 3a (KIF3A) is elevated in bladder cancer and can cause the occurrence of bladder cancer [12]. Through starBase and miRDB database prediction, there were binding sites between miR-139-5p and KIF3A. miR-145-5p has been discovered to reduce KIF3A expression [13]. Moreover, KIF3A can inhibit the expression of p21 [14]. p21 belongs to the family of cyclin-dependent kinase inhibitors, which regulate the cell cycle by inactivating cyclin-dependent kinase, a key regulator of the cell cycle [15]. The expression of p21 is downregulated in bladder cancer, and its up-regulation is able to delay bladder cancer [16, 17]. Therefore, this study aims at investigating the molecular mechanism of miR-139-5p delivered by BMSCs-EVs attenuating bladder cancer through the KIF3A/p21 axis.

## Materials and methods

### Ethics statement

The study protocol was approved by the Clinical Ethics Committee of Sichuan Academy of Medical Sciences & Sichuan Provincial People’s Hospital (approval number: 2012059) and conducted in line with the *Declaration of Helsinki*. Experiments involving animals were approved by the Animal Ethics Committee of Sichuan Academy of Medical Sciences & Sichuan Provincial People’s Hospital (approval number: 2019073) and performed in accordance with the Guide for the Care and Use of Laboratory Animals published by the US National Institutes of Health.

### Bioinformatics analysis

The miRNA expressed in the BMSCs-EVs was obtained through the EVmiRNA database. Bladder cancer-related miRNA expression dataset GSE40355 was retrieved from the GEO database. The dataset contains 16 bladder cancer samples and 8 normal samples. With |logFC| > 4 and *p* value < 0.01 as the threshold, R language “limma” package was operated for differential analysis of the GSE40355 dataset. The EVmiRNA database query results were intersected with the significantly downregulated miRNAs in bladder cancer samples using the Venn website. Finally, the starBase and miRDB databases were searched for predicting the binding sites between miR-139-5p and KIF3A.

### Tissue sample collection and BMSC isolation

Cancer tissues and adjacent normal tissues were collected from 75 patients with bladder cancer who underwent urethral bladder tumor resection at Sichuan Academy of Medical Sciences & Sichuan Provincial People’s Hospital from 2012 to 2015. The samples were all pathologically confirmed. None of the patients received preoperative chemotherapy or biotherapy. The samples were from 56 males and 19 females, with an average age of 61.72 (54–69 years; the median age was 62 years). Patients were followed up by means of outpatient data inquiry, outpatient, and telephone with a follow-up time of 5 years (from the day of a definite diagnosis to the patient’s death). The survival rate of the patients was analyzed by the Kaplan–Meier method.

Bone marrow samples were obtained from three normal human donors (one male and two females; the average age of 27 ± 2 years). The nucleated cells were isolated by the Percoll (1.073 g/mL) density gradient centrifugation method, seeded in a T25 culture plate with a density of 1 × 10^5^ cells/cm^2^ and cultured in a stem cell culture medium (Cyagen Biosciences Inc, Guangzhou, China) in an incubator (Thermo Fisher Scientific, Waltham, MA) at 37 °C and 5% CO_2_. The medium was changed every 3 days after the initial culture. When reaching 80–90% confluence, the BMSCs were treated with 0.25% trypsin (Gibco Life Technologies, Grand Island, NY) and prepared for subculture. Tissue samples were rapidly frozen in liquid nitrogen and stored at −80 °C.

### Identification of BMSCs

The morphology of BMSCs was observed under a light microscope. The BMSCs at passages 3–7 were cultured in OriCell^TM^ BMSC osteogenic, adipogenic or chondrogenic differentiation medium (Cyagen Biosciences Inc), followed by identification utilizing alizarin red staining, oil red o, and alcian blue staining respectively.

### Isolation of BMSCs-EVs

Logarithmically growing BMSCs were seeded into stem cell culture medium with 10% EV-free fetal bovine serum (FBS) (SA102.02, CellMax Life, San Diego, Califonia) and incubated in a cell incubator at 37 °C and 5% CO_2_. After 24 h, the supernatant of BMSCs was collected and centrifuged (300 × *g*, for 10 min; 2000 × *g* for 20 min) to remove the cells and cell fragments. Following three times of ultracentrifugation (XPN100, Beckman Coulter, Brea, CA) (100,000 × *g* for 90 min, for 90 min, and for 120 min), EVs were obtained, re-suspended in 100 μL sterile PBS and stored at −80 °C for later use. Information about EVs in this study has been submitted to EV track (https://www.evtrack.org) with the reference number of EV220156.

### Transmission electron microscope (TEM)

EVs were fixed in 100 mM PBS with 4% paraformaldehyde and 4% glutaraldehyde at ambient temperature. The fixed EVs were then dropped into the carbon-coated copper mesh and soaked in 2% phosphotungstic acid solution for 30 s. The observation was performed using a TEM (JEM-1010, JEOL, Tokyo, Japan).

### Nanoparticle tracking analysis (NTA)

The collected EVs were diluted with PBS to a particle concentration of 10^6^/mL–10^9^/mL, and then the samples were absorbed with a 1 mL syringe and injected into NanoSight LM10 (Malvern Instruments, Malvern, UK) for detection and analysis.

Information acquisition settings were referenced to a previous study [18]. First, a microsphere standard (known mass, density and concentration) similar to EVs was selected. After the microsphere was loaded, the camera level (shutter speed and gain were changed) was gradually increased until the image was close to saturation, and then slowly decreased until the microsphere was observed to be a single bright spot, with the focus adjusted if necessary. The microspheres should not blur or generate diffraction rings. There were ~20–60 microspheres per field at a concentration of ~2–10 × 10^8^/mL. Finally, the parameters of 20 ms shutter speed and 500 camera gain were used for data acquisition, and a 30 s video was shot. A new sample size was loaded and another record was performed. This procedure was repeated until five videos were captured. The settings remained the same between samples, and the sample tank was required to be cleaned when changing samples. Finally, each video was analyzed using NTA 2.3 software to give an estimate of the average EV size as well as the EV concentration.

### Cell culture treatment

Human normal bladder epithelial cell SV-HUC-1 (CL-0222, Wuhan Procell Life Science & Technology Co., Ltd., Wuhan, China) was cultured in F12K medium (19-0080-500, Tiandz, Beijing, China) appended to 10% FBS, 100 U/mL penicillin and 100 μg/mL streptomycin (C0222, Beyotime Institute of Biotechnology, Jiangsu, China). Human bladder cancer cell lines T24 (CL-0227, Procell) and BOY-12E (GD-C00513706S, Shanghai Guan&Dao Biological Engineering Co., Ltd., Shanghai, China) were cultured in RPMI-1640 medium (10-040-CVR, Corning Inc., Corning, NY) appended to 10% EV-free FBS, 100 U/mL penicillin, and 100 g/mL streptomycin. The aforementioned cells were cultured in a saturated humidity incubator at 37 °C and 5% CO_2_, respectively. The culture medium was discarded, and the cells were washed twice with PBS and digested by 0.25% trypsin for 2–5 min. T24 and BOY-12E cells were re-suspended in 5 mL complete medium for subculture.

T24 and BOY-12E cells with 55–80% confluence were transfected with negative control (NC) mimic, miR-139-5p mimic, NC inhibitor, miR-139-5p inhibitor, oe-NC, oe-KIF3A, si-NC, si-KIF3A, and si-p21 utilizing lipo2000 mixture (11668027, Thermo Fisher Scientific). About 48 h after transfection, the cells were collected, and RNA and protein were extracted for subsequent experiments.

### Lentivirus infection

The BMSCs were infected with lentivirus expressing NC mimic, miR-139-5p mimic, NC inhibitor and miR-139-5p inhibitor. The green fluorescent protein (GFP)-labeled puromycin-resistant miR-139-5p lentiviral overexpression vector, miR-139-5p lentiviral silencing vector with a virus titer of 2 × 10^10^ TU/mL (the lentiviral plasmid vector was pCDH-CMV-EF1-copGFP) (both purchased from Genechem, Shanghai, China) were selected to infect logarithmically growing BMSCs which were then incubated with 10 μg/mL Polybrene (H8761, Beijing Solarbio Science & Technology Co., Ltd, Beijing, China) in a 5% CO_2_ incubator at 37 °C. After 48–72 h of infection, BMSCs were placed in a medium containing 0.5 μg/mL puromycin (A1113803, Invitrogen, Carlsbad, CA). After 12 days, all BMSCs without infection died, and those with infections still survived. After that, the cell lines with stable expression were obtained by expanding the culture under the pressure of 300 mg/L.

### Internalization of EVs by bladder cancer cells

According to the instructions of PKH67 green fluorescent cell linker Kit (PKH67GL, Sigma Aldrich, St. Louis, Missouri), the EVs were subjected to fluorescence labeling. EVs were washed with PBS and centrifuged at 100,000 × *g* at 4 °C for 20 min to collect EVs. Next, EVs were labeled with PKH67 and co-cultured with T24 and BOY-12E cells at a concentration of 100 μg/mL in an incubator with 5% CO_2_ and saturated humidity at 37 °C. After 48 h of culture, the fluorescence imaging of PKH67 was observed under a fluorescence microscope (EVOS M5000, Thermo Fisher Scientific). PBS was used as a NC.

### Internalization of miR-139-5p delivered by BMSCs-EVs by bladder cancer cells

Initially, 1 × 10^5^ BMSCs were transfected with 15 nM Cy3-labeled miR-139-5p mimic (Genechem). After transfection for 24 h, 1 × 10^5^ BMSCs were placed in a stem cell culture medium. The EVs were extracted from BMSC medium 2 days later. The EVs were co-cultured with T24 and BOY-12E cells on a 0.4 μm polycarbonate filter plate (Corning Inc). After 12 h, the cells were washed twice with PBS, and then fixed in 2% paraformaldehyde for analysis employing a fluorescence microscope (BX53, Olympus Corporation, Japan).

### Cell Counting kit-8 (CCK-8) assay

Cell proliferation was detected with the CCK-8 kit (WH5199, Biotechwell, Shanghai, China). Logarithmically growing cells were cultured in RPMI-1640 medium appended to 10% FBS with concentration adjusted to 5 × 10^4^ cells/mL. Next, 100 μL cells were added to each well of a 96-well culture plate and 10 μL CCK-8 solution was added following at the corresponding time point for 2-h of incubation. Subsequently, five parallel wells were prepared for each group with the average value taken.

### Transwell assay

Matrigel gel (356235, BD-Biocoat) preserved at −80 °C was taken out and pre-coated in the transwell chamber (50 μL/well) for the invasion assay, while the transwell chamber without matrigel gel was prepared for the migration assay [19]. Six fields were randomly selected for observation, photographing, and counting under an inverted microscope.

### Hoechst 33342 and propidium iodide (PI) staining

Logarithmically growing T24 and BOY-12E cells were cultured in a 6-well plate (5 × 10^5^ cells per well). The cells were then stained with 10 μL Hoechst 33342 solution at 37 °C in darkness for 10 min, and then stained with 5 μL PI at 25 °C in darkness for 15 min. Observation of stained cells were implemented under a fluorescence microscope.

### Dual-luciferase reporter assay

The binding sites of miR-139-5p on the 3′UTR of KIF3A were analyzed utilizing the biological prediction website starBase and miRDB, followed by verification employing the dual-luciferase reporter assay. The synthetic KIF3A wild-type (WT) and mutant (MUT) were co-transfected with miR-139-5p mimic or miR-139-5p inhibitor respectively into 293 T cells (ATCC; cultured in DMEM appended to 10% FBS, 100 U/mL penicillin and 100 μg/mL streptomycin in a 5% CO_2_ incubator at 37 °C). Following 48-h of transfection, luciferase activity was detected by use of a Luminometer TD-20/20 detector (E5311, Promega Corporation) employing a Dual Luciferase Reporter Assay System kit (Promega Corporation).

### Reverse transcription-quantitative polymerase chain reaction (RT-qPCR)

miRNA analysis: Total RNA Purification Micro Kit (35350, Norgen Biotek, St. Catharines, ON, Canada) was processed to separate the RNA from the EVs, and the RNA was recovered with 20 μL Elution Solution E and stored at −80 °C. RNA concentration and mass were determined by means of a NanoDrop ND-2000 spectrophotometer, and miRNAs were subsequently reverse-transcribed in accordance with the miScript II RT Kit (218160, Qiagen GmbH, Hilden, Germany). Quantitative PCR detection was carried out in line with miScript SYBR® Green PCR Kit (218073, Qiagen GmbH, Hilden, Germany).

mRNA analysis: Total RNA was extracted from cultured cells employing Trizol reagent (15596026, Invitrogen), and then 2 μg total RNA was synthesized into cDNA referring to TaqMan reverse transcription Reagents (4304134, Invitrogen). RT-qPCR was operated utilizing the StepOnePlus™ real-time PCR System (4376600, Applied Biosystems, Thermo Fisher Scientific).

The primer sequence is described in Supplementary Table 1. miR-139-5p used cel-miR-39 and U6 as an internal reference, and other genes used β-actin as an internal reference. The 2^−ΔΔCt^ method was utilized to calculate the relative transcription level of target gene.

### Western blot analysis

Cells or tissues were added with 50 μL radio-immunoprecipitation assay (RIPA) lysis buffer (containing protease inhibitor) (P0013J, Beyotime, Shanghai, China) and protein was gained and quantified by utilization of the bicinchoninic acid (BCA) kit (PC0020, Solarbio). Later, 50 μg of protein was subjected to electrophoresis, and then transferred to nitrocellulose membranes (66485, Pall Corp, East Hills, NY). The membrane was sealed in 5% skimmed milk powder at ambient temperature for 2 h and probed with primary antibodies against KIF3A (ab11259, 1:2000, Abcam), p21 (ab109520, 1:1000, Abcam), p-p21 (ab47300, 1:1000, Abcam), Bax (ab32503, 1:1000, Abcam), Bcl-2 (#4223, 1:1000, Cell Signaling Technology [CST]), Cle-caspase 3 (ab32042, 1:500, Abcam), CD63 (ab134045, 1:1000, Abcam), CD81 (#56039, 1:1000, CST), Hsp70 (#4876, 1:1000, CST), Calreticulin (ab92516, 1:1000, Abcam) and β-actin (#4970, 1:1000, CST) overnight at 4 °C. The next day, the membrane was further incubated with horseradish peroxidase (HRP)-labeled secondary antibody goat anti-rabbit IgG (ab6721, 1:2000, Abcam) at ambient temperature for 1 h. Afterwards, the membrane was visualized by ECL reagent (BM101, Biomiga Inc), and then BioSpectrum 600 imaging system (Ultra-Violet Products, UK) was processed for detection and analysis. β-actin was used as normalizer.

### Nude mouse model with subcutaneous transplantation

BALB/c male nude mice (aged 8–12 weeks; Beijing Vital River Laboratory Animal Technology Co., Ltd., Beijing, China) were housed individually in the SPF laboratory at 22–25 °C and 60–65% humidity under a 12-h light/dark cycle (drink and eat freely). The mice were acclimated for one week before experiment.

Fifty nude mice were subcutaneously inoculated with T24 cells (1 × 10^6^), and 10 were randomly selected by computer as control group. The tumor diameter was measured utilizing vernier caliper every 3 days for the tumor volume calculation. When the tumor volume reached 50 mm^3^, the remaining 40 bladder cancer nude mice were injected via tail vein with lentiviral vectors expressing EVs-NC inhibitor (25 μg), EVs-miR-139-5p-inhibitor (25 μg), EVs-miR-139-5p-inhibitor + sh-NC, and EVs-miR-139-5p-inhibitor + sh-KIF3A. The EVs were injected every 3 days for 3 weeks. Tumor volume was measured every 3 days. When the weight of the nude mouse increased by more than 10% or the tumor volume exceeded 1000 mm^3^, the nude mouse was euthanized, the tumor was carefully excised and weighed; part of the tumor tissue was stored in liquid nitrogen, and the rest of the tumor was fixed with 10% formalin and paraffin-embedded for extraction of total RNA and protein.

### Lung metastasis model

Fifty 8–12-week-old BALB/c male nude mice were injected with 1 × 10^6^ T24 cells through the lateral tail vein. Among them, 10 were randomly selected as the control group, from which 5 of 10 nude mice were injected with PKH26-labeled EVs. After 48 h, the localization of EVs in lung tissue was observed with fluorescence microscope. The remaining 40 nude mice were treated the same as nude mouse model with subcutaneous transplantation. EVs were injected every 3 days. After 6 weeks of injection, mice were euthanatized, lung tissues were fixed with 4% paraformaldehyde, paraffin-embedded, and lung metastatic nodules were observed by hematoxylin-eosin (HE) staining.

### HE staining

First, 4-μm-thick lung tissue sections were deparaffinized in xylene, rehydrated in ethanol, and stained according to the HE staining kit (C0105, Beyotime Institute of Biotechnology). The lung metastasis nodules were observed under an optical microscope (CX41-12C02, Olympus Corporation, Japan), and the INFINITY ANALYZE software (Lumenera Corporation, Ottawa, Canada) was conducted to analyze the image.

### Statistical analysis

Statistical analysis was conducted by SPSS 21.0 (IBM Corp., Armonk, New York). Measurement data were described as mean ± standard deviation. Paired *t* test was conducted to compare the data between the cancer tissues and adjacent normal tissues. Unpaired *t* test was started for two-group data comparison. One-way analysis of variance (ANOVA) was processed for multi-group data comparison, followed by Tukey’s post hoc test. The Kaplan–Meier method was employed to calculate the survival rate of patients. Statistical significance was set up at *p* < 0.05, *p* < 0.01, *p* < 0.001 or *p* < 0.0001.

## Results

### BMSCs-EVs inhibit the malignant properties of bladder cancer cells

A study has shown that BMSCs-EVs can inhibit the proliferation of T24 cells by blocking the cell cycle of T24 cells, and induce T24 cell apoptosis [7]. Therefore, we first isolated BMSCs. We observed that BMSCs were long shuttle or spindle-shaped, colony growth, and had good osteogenic, adipogenic, and chondrogenic differentiation functions (Supplementary Fig. 1A). These results demonstrated the successful isolation of BMSCs. The EVs were then isolated from BMSCs, and a group of circular or elliptic membranous vesicles with relatively obvious heterogeneity in size and basically uniform morphology were observed under a TEM. Membranous structures were seen around the vesicles, with low electron density components in the center (Supplementary Fig. 1B). The results of NTA displayed that the EVs showed irregular Brownian motion with a diameter of 60–450 nm and an average diameter of 142 nm (Supplementary Fig. 1C). Western blot analysis results showed that surface markers of EVs including CD63, CD81, and Hsp70 were highly expressed, while Calreticulin was poorly expressed in the BMSCs-EVs (Supplementary Fig. 1D), which further suggested the successful extraction of EVs.

**Fig. 1:**
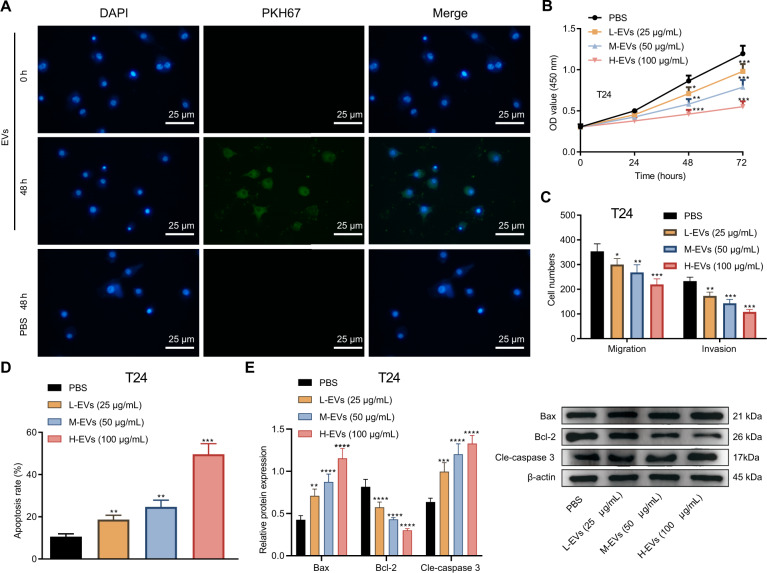
BMSCs-EVs prevent the proliferation, migration, and invasion of T24 cells while inducing their apoptosis. **A** The uptake of EVs labeled with PKH67 (green fluorescence) by the T24 cells analyzed using a fluorescence microscope. DAPI stained nucleus is blue. Scale bar: 25 μm. **B** The proliferation of T24 cells co-cultured with different concentrations of EVs detected by CCK-8. **C** The migration and invasion of T24 cells co-cultured with different concentrations of EVs detected using Transwell assay. **D** The apoptosis of T24 cells co-cultured with EVs with different concentrations tested using Hoechst 33342/PI staining. **E** The expression of apoptosis-related factors in T24 cells co-cultured with different concentrations of EVs detected using western blot analysis. Measurement data were expressed as mean ± standard deviation. Unpaired *t* test was performed for two-group data comparison. *p < 0.05, **p < 0.01, ***p < 0.001 or ****p < 0.0001, vs. PBS-treated T24 cells. The cell experiment was repeated three times independently.

In order to verify whether the BMSCs-EVs can enter bladder cancer cells and exert their biological functions, we co-cultured PKH67-labeled EVs with T24 and BOY-12E cells for 48 h. We identified green fluorescence in T24 and BOY-12E cells (Fig. 1A, Supplementary Fig. 2A), indicating that EVs labeled with PKH67 can be internalized by T24 and BOY-12E cells.

Then, different concentrations of EVs were co-cultured with T24 and BOY-12E cells. It was evident that as the concentration of EVs increased, the proliferation (Fig. 1B, Supplementary Fig. 2B), migratory and invasive features of T24 and BOY-12E cells (Fig. 1C, Supplementary Fig. 2C) were progressively attenuated. In addition, the results of Hoechst 33342/PI staining and Western blot demonstrated a progressive increase in the apoptosis of T24 and BOY-12E cells (Fig. 1D, Supplementary Fig. 2D), accompanied by decreased protein expression of Bcl-2 (Fig. 1E, Supplementary Fig. 2E) and elevated protein expression of Bax and Cle-caspase 3 (Fig. 1E, Supplementary Fig. 2E) as the concentration of EVs increased. To sum up, BMSCs-EVs can inhibit the malignant features of bladder cancer cells.

### BMSCs-EVs transfer miR-139-5p into bladder cancer cells

**Fig. 2:**
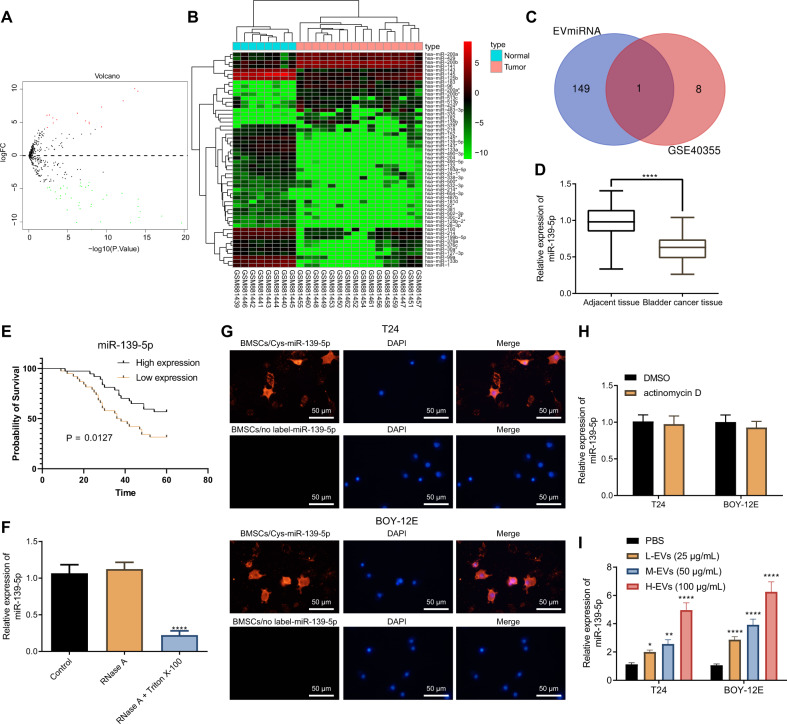
BMSCs-EVs deliver miR-139-5p into bladder cancer cells. **A** A volcano map of miRNA differential expression in the GSE40355 dataset. The black part indicates the miRNA with no significant difference in bladder cancer, the green represents the miRNA that was significantly downregulated in bladder cancer, and the red represents the miRNA that was significantly upregulated in bladder cancer. **B** A heat map of expression of the 15 most significantly upregulated and 39 most downregulated miRNAs in the GSE40355 dataset. **C** Screening of miRNAs that were significantly downregulated in bladder cancer and expressed in BMSCs-EVs. The left circle represents the top 150 miRNAs of EVmiRNA query results, the right circle represents the top 10 miRNAs significantly downregulated in GSE40355 dataset, and the middle part represents the intersection of the two groups of data. **D** The expression of miR-139-5p in bladder cancer tissues and the adjacent normal tissues measured using RT-qPCR (*n* = 75). ****p < 0.0001. **E** The relationship between the survival rate of bladder cancer patients and miR-139-5p expression in bladder cancer tissues analyzed by the Kalpan-Meier method (*n* = 75). **F** The expression of miR-139-5p in the BMSC culture medium after the addition of RNase A alone or RNase A and Triton X-100 co-addition to treat EVs tested using RT-qPCR. ****p < 0.0001. **G** The uptake of Cy3-labeled BMSCs-EVs-miR-139-5p (red fluorescence) by T24 and BOY-12E cells was observed by a fluorescence microscope. The cytoplasm stained with DAPI shows blue (Scale bar = 50 μm). **H** The expression of miR-139-5p in T24 and BOY-12E cells after actinomycin D treatment monitored by RT-qPCR. **I** The expression of miR-139-5p in T24 and BOY-12E cells co-cultured with different concentrations of EVs detected by RT-qPCR. The measurement data were expressed as mean ± standard deviation. Paired *t* test was conducted to compare data between the cancer tissues and adjacent normal tissues. Unpaired *t* test was performed for a two-group data comparison. One-way ANOVA was conducted for multi-group data comparison, followed by Tukey’s post hoc test. *p < 0.05, **p < 0.01, or ****p < 0.0001, vs. PBS group or RNase A group. The cell experiment was repeated three times independently.

Differential analysis of the bladder cancer-related miRNA expression dataset GSE40355 revealed 15 significantly upregulated and 39 significantly downregulated in the bladder cancer samples (Fig. 2A). The R language “pheatmap” package was processed to visualize these miRNAs in bladder cancer tissues (Fig. 2B). The miRNAs expressed in the BMSCs-EVs were obtained using EVmiRNA database, of which the first 150 miRNAs were intersected with the first 10 miRNAs significantly downregulated in the bladder cancer samples in the GSE40355 dataset, with miR-139-5p found at the intersection (Fig. 2C). A previous study has shown a reduction in miR-139-5p expression in bladder cancer [11]. Therefore, miR-139-5p was selected as the target for the subsequent assay.

A decline of miR-139-5p was found in bladder cancer tissues (Fig. 2D). Kaplan–Meier curve analysis results suggested that the survival rate of bladder cancer patients was significantly higher in the miR-139-5p high expression group than that in the miR-139-5p low expression group (Fig. 2E).

For verifying whether BMSCs-EVs carry miR-139-5p and can deliver miR-139-5p to bladder cancer cells, we first treated the BMSCs-EVs with RNase A alone or with RNase A and Triton X-100 simultaneously. The results of RT-qPCR displayed that further Triton X-100 administration led to decreased miR-139-5p expression under RNase A treatment (Fig. 2F). This indicated the presence of miR-139-5p in BMSCs-EVs.

Subsequently, Cy3-labeled miR-139-5p mimic was transfected into BMSCs, and the EVs were isolated and co-cultured with T24 and BOY-12E cells. After 24 h, Cy3-miR-139-5p red fluorescence was observed in the cytoplasm of T24 and BOY-12E cells (Fig. 2G). At the same time, to rule out the possibility that the expression of miR-139-5p was endogenously induced, we used actinomycin D to treat T24 and BOY-12E cells. It was found that the expression of miR-139-5p showed no significant changes in T24 and BOY-12E cells after treatment with actinomycin D compared with DMSO treatment (Fig. 2H). Finally, EVs of different concentrations were co-cultured with T24 and BOY-12E cells. We found that with the increase of EVs concentration, the expression of miR-139-5p in T24 and BOY-12E cells showed an increasing trend (Fig. 2I). In summary, miR-139-5p was present in the BMSCs-EVs and could be transferred into bladder cancer cells by BMSCs-EVs.

### miR-139-5p delivered by BMSCs-EVs arrests malignant features of bladder cancer cells

**Fig. 3:**
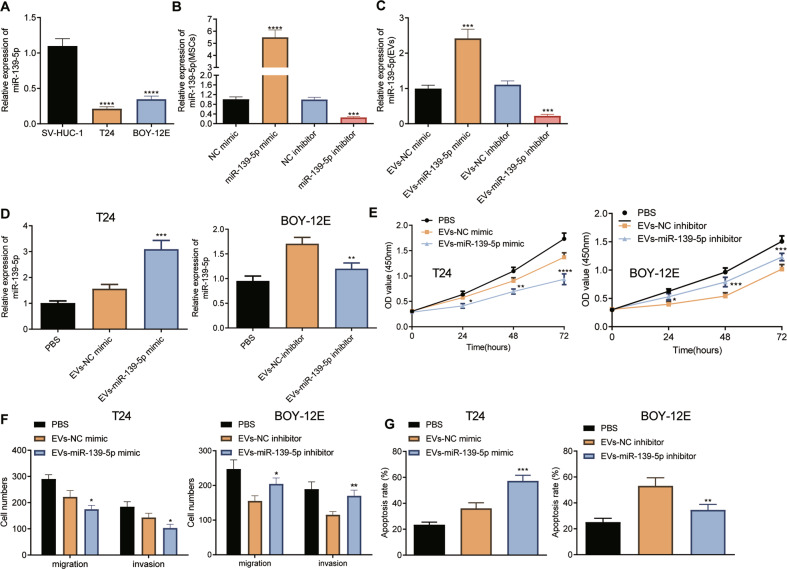
miR-139-5p delivered by BMSCs-EVs represses the proliferation, migration, and invasion of bladder cancer cells while inducing their apoptosis. **A** The expression of miR-139-5p in SV-HUC-1, T24, and BOY-12E cells detected using RT-qPCR. ****p < 0.0001, vs. SV-HUC-1 cells. **B** The expression of miR-139-5p in BMSCs treated with miR-139-5p mimic or miR-139-5p inhibitor measured using RT-qPCR. ***p < 0.001 or ****p < 0.0001, vs. NC mimic or NC inhibitor. **C** The expression of miR-139-5p in the BMSCs-EVs treated with EVs-miR-139-5p mimic or EVs-miR-139-5p inhibitor measured using RT-qPCR. **D** The expression of miR-139-5p in T24 cells co-cultured with EVs-miR-139-5p mimic, and BOY-12E cells co-cultured with EVs-miR-139-5p inhibitor detected using RT-qPCR. **E** The proliferation of T24 cells co-cultured with EVs-miR-139-5p mimic, and BOY-12E cells co-cultured with EVs-miR-139-5p inhibitor detected using RT-qPCR. **F** Migration and invasion of T24 cells co-cultured with EVs-miR-139-5p mimic, and BOY-12E cells co-cultured with EVs-miR-139-5p inhibitor detected using Transwell assay. **G** Apoptosis of T24 cells co-cultured with EVs-miR-139-5p mimic, and BOY-12E cells co-cultured with EVs-miR-139-5p inhibitor detected using Hoechst 33342/PI staining. Measurement data were expressed as mean ± standard deviation. One-way ANOVA was conducted for multi-group data comparison, followed by Tukey’s post hoc test. In panels (**C**–**G**), *p < 0.05, **p < 0.01, ***p < 0.001 or ****p < 0.0001, vs. EVs-NC mimic or EVs-NC inhibitor. The cell experiment was repeated three times independently.

We then study the effect of miR-139-5p delivered by BMSCs-EVs on the biological function of bladder cancer cells. The expression of miR-139-5p was diminished in both T24 and BOY-12E cells than that in SV-HUC-1 cells (Fig. 3A). Then miR-139-5p was over-expressed or silenced in BMSCs, RT-qPCR found that the expression of miR-139-5p was increased by about nine times in miR-139-5p mimic-transduced BMSCs, whereas it was reduced by ~74.32% following miR-139-5p inhibitor (Fig. 3B). Following extraction of EVs, we observed that miR-139-5p expression was elevated upon EVs-miR-139-5p mimic; however, EVs-miR-139-5p inhibitor led to a contrary result (Fig. 3C). Since the expression of miR-139-5p in BOY-12E cells was higher than that in T24 cells (Fig. 3A). Therefore, the T24 cells were co-cultured with EVs-miR-139-5p mimic, and BOY-12E cells were co-cultured with EVs-miR-139-5p inhibitor. RT-qPCR results showed that the expression of miR-139-5p was elevated in T24 cells co-cultured with EVs-miR-139-5p mimic, while it was decreased in BOY-12E cells co-cultured with EVs-miR-139-5p inhibitor (Fig. 3D). Furthermore, EVs-miR-139-5p mimic suppressed the proliferative (Fig. 3E), migratory and invasive potentials (Fig. 3F) of T24 cells, but promoted the apoptosis (Fig. 3G). On the contrary, EVs-miR-139-5p inhibitor in BOY-12E cells reversed the aforementioned results (Fig. 3E–G). Altogether, miR-139-5p in the BMSCs-EVs can suppress the malignant potentials of bladder cancer cells.

### miR-139-5p in BMSCs-EVs targets and inhibits KIF3A

**Fig. 4:**
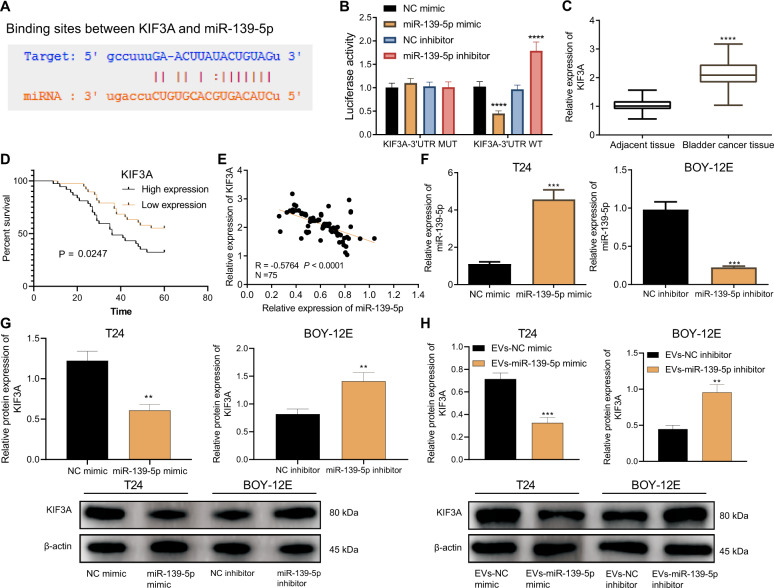
miR-139-5p delivered by BMSCs-EVs targets KIF3A and inhibits KIF3A expression. **A** Binding sites between KIF3A and miR-139-5p predicted by the starBase and miRDB databases. **B** Binding of miR-139-5p to KIF3A verified using dual-luciferase reporter assay. ****p < 0.0001, vs. NC mimic or NC inhibitor. **C** The expression of KIF3A in bladder cancer tissues and adjacent normal tissues detected using RT-qPCR (*n* = 75). ****p < 0.0001, vs. adjacent normal tissues. **D** The relationship between the survival rate of patients with bladder cancer and KIF3A expression analyzed by the Kaplan–Meier method (*n* = 75). **E** Correlation between miR-139-5p expression and KIF3A expression in cancer tissues of patients with bladder cancer (*n* = 75) analyzed by Pearson’s correlation coefficient. **F** The expression of miR-139-5p in T24 cells transfected with miR-139-5p mimic and BOY-12E cells transfected with miR-139-5p inhibitor detected by RT-qPCR. **G** The expression of KIF3A in T24 cells transfected with miR-139-5p mimic and BOY-12E cells transfected with miR-139-5p inhibitor detected by western blot analysis. In panels (**F**, **G**), **p < 0.01 or ***p < 0.001 vs. NC mimic or NC inhibitor. **H** KIF3A expression in T24 cells co-cultured with EVs-miR-139-5p mimic and BOY-12E cells co-cultured with EVs-miR-139-5p inhibitor tested by western blot analysis. **p < 0.01 or ***p < 0.001 vs. EVs-NC mimic or EVs-NC inhibitor. Measurement data were expressed as mean ± standard deviation. Paired *t* test was conducted to compare data between the cancer tissues and adjacent normal tissues. Unpaired *t* test was performed for two-group data comparison. The cell experiment was repeated three times independently.

Existing literature has shown that KIF3A is highly expressed in bladder cancer and can promote the occurrence of bladder cancer [12]. The starBase and miRDB databases predicted the presence of binding sites between miR-139-5p and KIF3A (Fig. 4A). Therefore, we examined whether miR-139-5p can affect the development of bladder cancer by targeting KIF3A. Luciferase activity of miR-139-5p mimic + KIF3A-3~UTR WT was repressed while that of miR-139-5p inhibitor + KIF3A-3′UTR WT was increased. No alterations were found in the luciferase activity of miR-139-5p mimic + KIF3A-3′UTR MUT or miR-139-5p inhibitor + KIF3A-3’UTR MUT (Fig. 4B). These data suggested that miR-139-5p can target KIF3A. The results of RT-qPCR detected that KIF3A was elevated in bladder cancer tissues (Fig. 4C). The Kaplan–Meier method revealed that the survival rate of bladder cancer patients in the KIF3A high expression group was lower than that in the KIF3A low expression group (Fig. 4D). At the same time, an adverse correlation was detected between miR-139-5p and KIF3A expression in bladder cancer tissues (Fig. 4E).

To pinpoint whether miR-139-5p can modulate KIF3A in bladder cancer cells, we first over-expressed miR-139-5p in T24 cells, and silenced miR-139-5p in BOY-12E cells. It was exhibited that miR-139-5p expression in T24 cells transfected with miR-139-5p mimic increased by about seven times, while it was reduced by about 77.17% in BOY-12E cells transfected with miR-139-5p inhibitor (Fig. 4F). Afterward, a decline in KIF3A expression was detected in T24 cells transfected with miR-139-5p mimic, while an elevation was seen in BOY-12E cells transfected with miR-139-5p inhibitor (Fig. 4G). In addition, EVs-miR-139-5p mimic induced reduced KIF3A expression in T24 cells but EVs-miR-139-5p-inhibitor elevated KIF3A expression in BOY-12E cells (Fig. 4H). In summary, miR-139-5p delivered by BMSCs-EVs targeted KIF3A and suppressed KIF3A expression.

### miR-139-5p targets KIF3A to impair the malignant potentials of bladder cancer cells

**Fig. 5:**
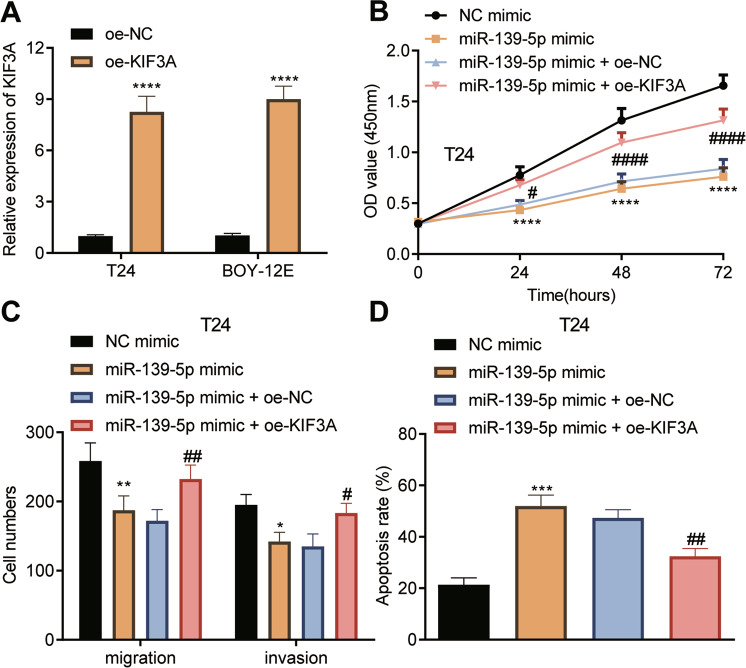
miR-139-5p targets KIF3A to alleviate the proliferation, migration, and invasion of T24 cells while inducing their apoptosis. **A** The expression of KIF3A in T24 and BOY-12E cells transfected with oe-KIF3A detected by RT-qPCR. ****p < 0.0001, vs. oe-NC. **B** The proliferation of T24 cells transfected with miR-139-5p mimic or combined with oe-KIF3A detected using CCK-8. **C** The migration and invasion of T24 cells transfected with miR-139-5p mimic or combined with oe-KIF3A monitored using Transwell. **D** The apoptosis of T24 cells transfected with miR-139-5p mimic or combined with oe-KIF3A detected by Hoechst 33342/PI staining. *p < 0.05, **p < 0.01, ***p < 0.001 or ****p < 0.0001, vs. NC mimic. ^#^*p* < 0.05, ^##^*p* < 0.01 or ^####^*p* < 0.0001, vs. miR-139-5p mimic + oe-NC. Measurement data were expressed as mean ± standard deviation. Unpaired *t* test was performed for two-group data comparison. One-way ANOVA was conducted for multi-group data comparison, followed by Tukey’s post hoc test. The cell experiment was repeated three times independently.

In the following, T24 and BOY-12E cells were transfected with oe-KIF3A or/and miR-139-5p mimic. As described by RT-qPCR, KIF3A expression was elevated approximately eight times in T24 and BOY-12E cells in the presence of oe-KIF3A (Fig. 5A). In addition, overexpression of miR-139-5p could limit T24 and BOY-12E cell proliferation (Fig. 5B, Supplementary Fig. 3A), migration and invasion (Fig. 5C, Supplementary Fig. 3B), and promote their apoptosis (Fig. 5D, Supplementary Fig. 3C). However, overexpression of KIF3A reversed these effects. Overall, miR-139-5p arrested the malignant potentials of bladder cancer cells through silencing KIF3A.

### miR-139-5p delivered by BMSCs-EVs downregulates KIF3A to activate p21, thus inhibiting the malignant potentials of bladder cancer cells

**Fig. 6:**
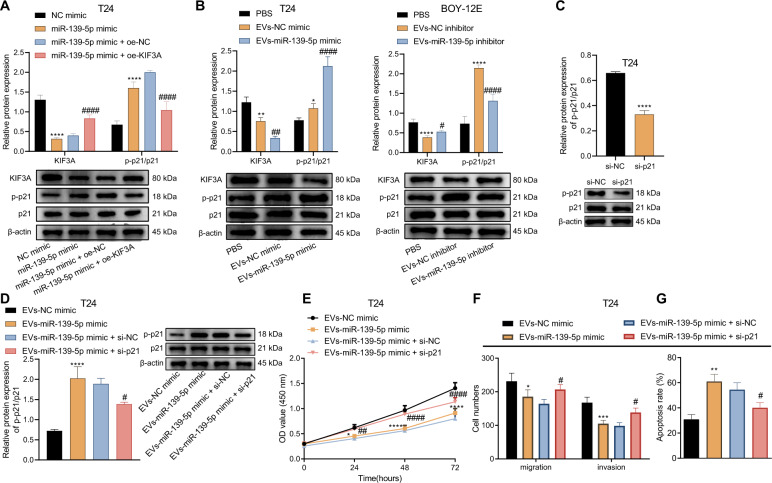
miR-139-5p delivered by BMSCs-EVs downregulates KIF3A to activate p21, thus inhibiting the proliferation, migration and invasion of T24 cells while inducing their apoptosis. **A** The expression of KIF3A and the ratio of p-p21/p21 in T24 cells transfected with miR-139-5p mimic or combined with oe-KIF3A detected by western blot analysis. ****p < 0.0001, vs. NC mimic. ^####^*p* < 0.0001, vs. miR-139-5p mimic + oe-NC. **B** The expression of KIF3A and ratio of p-p21/p21 in T24 cells co-cultured with EVs-miR-139-5p mimic and BOY-12E cells co-cultured with EVs-miR-139-5p inhibitor monitored by western blot analysis. *p < 0.05, **p < 0.01, or ****p < 0.0001, vs. PBS. ^#^*p* < 0.05, ^##^*p* < 0.01 or ^####^*p* < 0.0001, vs. EVs-NC mimic or EVs-NC inhibitor. **C** si-p21 transfection efficiency in T24 cells detected by western blot analysis. ****p < 0.0001, vs. si-NC. **D** p-p21/p21 ratio in T24 cells co-cultured with EVs-miR-139-5p mimic or combined with si-p21 detected by western blot analysis. **E** The proliferation of T24 cells co-cultured with EVs-miR-139-5p mimic or combined with si-p21 monitored by CCK-8. **F** The migration and invasion of T24 cells co-cultured with EVs-miR-139-5p mimic or combined with si-p21 monitored by Transwell assay. **G** The apoptosis of T24 cells co-cultured with EVs-miR-139-5p mimic or combined with si-p21 monitored by Hoechst 33342/PI staining. In panels D-G, *p < 0.05, **p < 0.01, ***p < 0.001, or ****p < 0.0001, vs. EVs-NC mimic. ^#^*p* < 0.05, ^##^*p* < 0.01, or ^####^*p* < 0.0001, vs. EVs-miR-139-5p mimic + si-NC. Measurement data were expressed as mean ± standard deviation. Unpaired *t* test was performed for two-group data comparison. One-way ANOVA was conducted for multi-group data comparison, followed by Tukey’s post hoc test. The cell experiment was repeated three times independently.

A prior study has shown that KIF3A can limit p21 expression [14], and p21 can inhibit the occurrence of bladder cancer [20, 21]. Therefore, we included p21 into the research scope. After overexpression of miR-139-5p in T24 and BOY-12E cells, the expression of KIF3A was decreased and the ratio of p-p21/p21 was increased, further overexpression of brought about contrary trends (Fig. 6A, Supplementary Fig. 4A). Western blot analysis results indicated that KIF3A expression was dropped in T24 cells co-cultured with EVs-miR-139-5p mimic but the ratio of p-p21/p21 was elevated. Opposite results were noted in the KIF3A expression and ratio of p-p21/p21 in BOY-12E cells co-cultured with EVs-miR-139-5p inhibitor (Fig. 6B).

Moreover, the expression of p21 was reduced in T24 and BOY-12E cells transfected with si-p21 (Fig. 6C, Supplementary Fig. 4B). Further, an increase in the p-p21/p21 ratio was detected in T24 and BOY-12E cells co-cultured with EVs-miR-139-5p mimic yet a decline was noted in the presence of EVs-miR-139-5p mimic + si-p21 (Fig. 6D, Supplementary Fig. 4C). Functionally, EVs-miR-139-5p mimic inhibited the proliferation (Fig. 6E, Supplementary Fig. 4D), migration, and invasion of T24 and BOY-12E cells (Fig. 6F, Supplementary Fig. 4E), and promote their apoptosis (Fig. 6G, Supplementary Fig. 4F). However, silencing of p21 reversed the effect of EVs-miR-139-5p mimic. In summary, the miR-139-5p delivered by BMSCs-EVs decreased KIF3A expression to activate p21, thus limiting the malignant potentials of bladder cancer cells.

### miR-139-5p delivered by BMSCs-EVs prevents the tumorigenesis and metastasis of bladder cancer cells in vivo by regulating the KIF3A/p21 axis

**Fig. 7:**
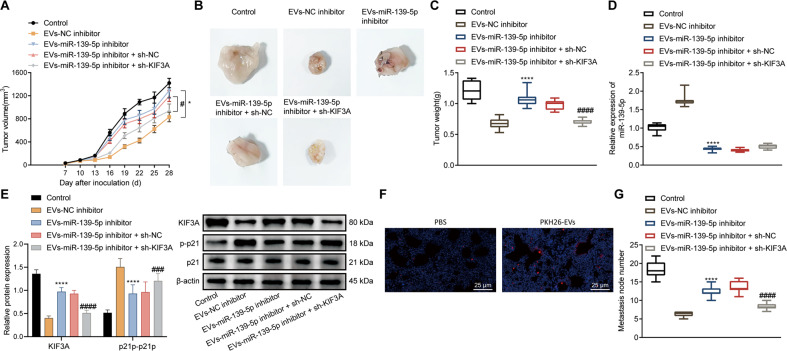
miR-139-5p delivered by BMSCs-EVs impedes the tumorigenesis and metastasis of bladder cancer cells in vivo by regulating the KIF3A/p21 axis. **A** Tumor volume of mice treated with EVs-miR-139-5p inhibitor or combined with sh-KIF3A. **B** Representative images showing xenografts in mice treated with EVs-miR-139-5p inhibitor or combined with sh-KIF3A. **C** Tumor weight of mice treated with EVs-miR-139-5p inhibitor or combined with sh-KIF3A. **D** miR-139-5p expression in tumor tissues of mice treated with EVs-miR-139-5p inhibitor or combined with sh-KIF3A detected using RT-qPCR. **E** The expression of KIF3A and ratio of p-p21/p21 in tumor tissues of mice treated with EVs-miR-139-5p inhibitor or combined with sh-KIF3A tested by western blot analysis. **F** The location of EVs labeled with PKH26 (red fluorescence) in the lung tissue of nude mice observed under a fluorescence microscope. Scale bar: 25 μm. **G** Lung metastatic nodules in mice treated with EVs-miR-139-5p inhibitor or combined with sh-KIF3A observed by HE staining. Measurement data were expressed as mean ± standard deviation, One-way ANOVA was conducted for multi-group data comparison, followed by Tukey’s post hoc test. Repeated measures ANOVA was used for comparison of data between multiple groups at different time points, followed by Bonferroni post hoc test. *p < 0.05 or ****p < 0.0001 vs. EVs-NC-inhibitor group. ^#^ <0.05 or ^###^*p* < 0.001, ^####^*p* < 0.0001, vs. EVs-miR-139-5p-inhibitor + sh-NC group. *n* = 10 for mice in each group.

For validation in vivo, we injected T24 cells subcutaneously into nude mice to establish a subcutaneous transplantation tumor model, and then injected EVs-miR-139-5p-inhibitor or combined with sh-KIF3A. The results showed that the EVs-miR-139-5p-inhibitor led to increased tumor volume (Fig. 7A, B) and weight (Fig. 7C), while further sh-KIF3A caused opposite trends. In addition, miR-139-5p expression and p-p21/p21 ratio were decreased and KIF3A expression was increased in the tumor tissues of EVs-miR-139-5p inhibitor-treated mice. In the presence of EVs-miR-139-5p-inhibitor + sh-KIF3A, miR-139-5p expression showed no changes, KIF3A expression was diminished and p-p21/p21 ratio was increased (Fig. 7D, E).

We then assessed the effect of miR-139-5p delivered by BMSCs-EVs on tumor metastasis, we also injected T24 cells into nude mice through the lateral tail vein to establish a lung metastasis model, followed by injection of PKH26-labeled EVs. PKH26 red fluorescence was observed in the lung tissue of nude mice 48 h later (Fig. 7F). HE staining results noted that mice treated with EVs-miR-139-5p-inhibitor exhibited enhanced lung metastasis ability, while further sh-KIF3A treatment caused a repressed phenomenon (Fig. 7G). In summary, the miR-139-5p delivered by BMSCs-EVs reduced KIF3A to activate p21, thus limiting the tumorigenesis and metastasis of bladder cancer cells in vivo.

## Discussion

Chemotherapy to remove residual cancer cells after the surgical intervention within the bladder is the standard treatment for bladder cancer [22]. However, high recurrence makes bladder cancer remains head-scratching [23]. Recently, targeted molecular therapies have been developed for bladder cancer [24]. EVs have been discovered to function importantly in the local tumor progression, metastasis, and spread of bladder cancer [25]. Our study here was intended to elucidate the molecular mechanism of miR-139-5p delivered by BMSCs-EVs in bladder cancer. Consequently, this study uncovered that BMSC-EV-miR-139-5p restrained bladder cancer from occurrence through the mediation of KIF3A/p21 axis.

At the beginning of our experiments, we found that BMSCs-EVs inhibited the malignant features of bladder cancer cells. EVs have been considered as a novel cell-free therapeutic strategy for the treatment of a great number of diseases and cancers [26]. Due to their natural potentials to regulate cell-to-cell communication and physicochemical properties, EVs are considered as excellent nanomedicine for drug transport and release in cancer therapy [27]. Meanwhile, accumulating evidence confirms that EVs can be modified or engineered to enhance their efficiency, safety, and specificity for cancer therapy [26, 28]. Especially, BMSCs-EVs are known to exert therapeutic effects in tissue regeneration, and have also been used as a gene transfer system for cellular therapy [29]. It has been reported that BMSCs-EVs can inhibit T24 cell proliferation by blocking the cell cycle of T24 cells, and induce T24 cell apoptosis [7]. Besides, bladder cancer cell-derived exosomes inhibit tumor cell apoptosis and promote cell proliferation in vitro, accompanied by elevated Bcl-2 expression and limited protein expression of Bax and caspase-3 with the increase of EV concentration [30]. The above literature results are consistent with our findings that the proliferation, migration and invasion ability together with Bcl-2 expression were reduced while apoptosis and Bax and Cle-caspase 3 expression were increased in T24 cells in an EV-dependent manner. Then, we found that miR-139-5p was present in the BMSCs-EVs and BMSCs-EVs transferred miR-139-5p to bladder cancer cells. BMSCs-EVs, loaded with miRNAs, have demonstrated inhibitory properties in glioblastomas [31], multiple myeloma [32], and oral cancer [33]. A previous report has confirmed miR-139-5p derived from circulating EVs as a non-invasive bio-marker for the diagnosis of lung cancer [34]. miR-139-5p expression is notably decreased in bladder cancer tissues and cells [35]. Furthermore, our study revealed that miR-139-5p delivered by BMSCs-EVs could inhibit the malignant features of bladder cancer cells. In line with our findings, human umbilical cord mesenchymal stem cells-derived exosomal miR-139-5p inhibits tumorigenesis, playing a suppressive role in the progression of bladder cancer both in vivo and in vitro [36]. Moreover, up-regulation of miR-139-5p can suppress the proliferation, migration, and invasion of bladder cancer cells [11]. Thus, combined with our results, we believe that BMSCs-EVs containing miR-139-5p has potential clinical value and may be applied to the treatment of bladder cancer in the future. However, we also recognize that clinical transformation is still a long process, but this study provides a good theoretical basis and research direction.

Subsequent results of the present study displayed that miR-139-5p targeted and inhibited KIF3A. As described previously, KIF3A is elevated in bladder cancer and can induce bladder cancer [12]. miR-145-5p has been discovered to inhibit the expression of KIF3A [13]. Later, the current results pinpointed that miR-139-5p targeted KIF3A and downregulated KIF3A expression to inhibit the malignant features of bladder cancer cells. Consistently, KIF3A accelerates the malignant capabilities of bladder cancer cells in vivo and in vitro [12]. In addition, KIF3A knockdown impairs the proliferation of triple-negative breast cancer cells by suppressing the Rb-E2F signaling pathway [14]. Then, it has been identified that miR-139-5p delivered by BMSCs-EVs decreased KIF3A expression to activate p21, thereby restraining the malignant behaviors of bladder cancer cells. Overexpression of miR-139 in prostate cancer cells can lead to a significant reduction in cell proliferation and migration and an elevation of p21 [37]. Meanwhile, activation of p21 is related to decreased proliferation, migration, and invasion of bladder cancer cells, which indicates a better prognosis for patients with bladder cancer [38]. At last, in vivo experimental results have shown that BMSCs-derived exosomal miR-139-5p downregulated KIF3A expression to retard the tumorigenesis and metastasis of bladder cancer cells in vivo.

**Fig. 8:**
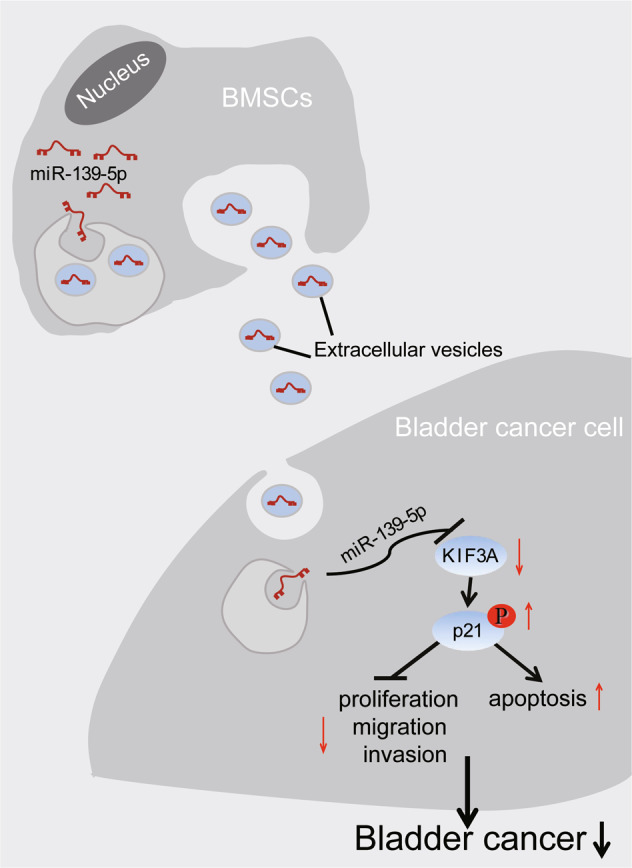
Molecular mechanisms of our study. Mechanism diagram showing that miR-139-5p delivered by BMSCs-EVs attenuates the progression of bladder cancer through suppression of KIF3A and activation of p21.

Taken together, miR-139-5p delivered by BMSCs-EVs could potentially attenuate the progression of bladder cancer through suppression of KIF3A and activation of p21 (Fig. 8). Our findings pinpoint a theoretical basis and shed light on the treatment of bladder cancer.

## Supplementary information

supplemental materials

aj-checklist

## Data Availability

The data and materials of the study can be obtained from the corresponding author upon request.
