# Supplementary material for: Tumor suppressive role of microRNA-139-5p in bone marrow mesenchymal stem cells-derived extracellular vesicles in bladder cancer through regulation of the KIF3A/p21 axis
Source: Cell Death Dis. 2022 Jul 12;13(7):599. doi: 10.1038/s41419-022-04936-0 (PMC9276749; doi:10.1038/s41419-022-04936-0)
Supplement: Supplementary file 1 — supplemental materials [file 41419_2022_4936_MOESM1_ESM.docx]

Supplementary Table 1 Primer sequences for RT-qPCR

| Target | Sequences |
| --- | --- |
| miR-139-5p | F: 5’-CGACGCGTCCCTCTTCCCATTCCTTC-3’ |
|  | R: Universal Reverse Primer (miScript II RT Kit) |
| U6 | F: 5’-CGCTTCGGCAGCACATA-3’ |
|  | R: 5’-Universal Reverse Primer (miScript II RT Kit)-3’ |
| KIF3A | F: 5’-TCCCGTTCCCATGCCATCTT-3’ |
|  | R: 5’-GCTTCCTTTAGGCGCTGTCC-3’ |
| β-actin | F: 5’-AGCGAGCATCCCCCAAAGTT-3’ |
|  | R: 5’-GGGCACGAAGGCTCATCATT-3’ |
| cel-miR-39 | F: 5’-GGTCACCGGGTGTAAATCAGCTTG-3’  R: Universal Reverse Primer (miScript II RT Kit) |

Note: miR-139-5p, microRNA-139-5p; U6, U6 snRNA; KIF3A, kinesin family member 3a; β-actin, Beta-actin; cel-miR-39, cel-microRNA-39; F, forward; R, reverse.

**
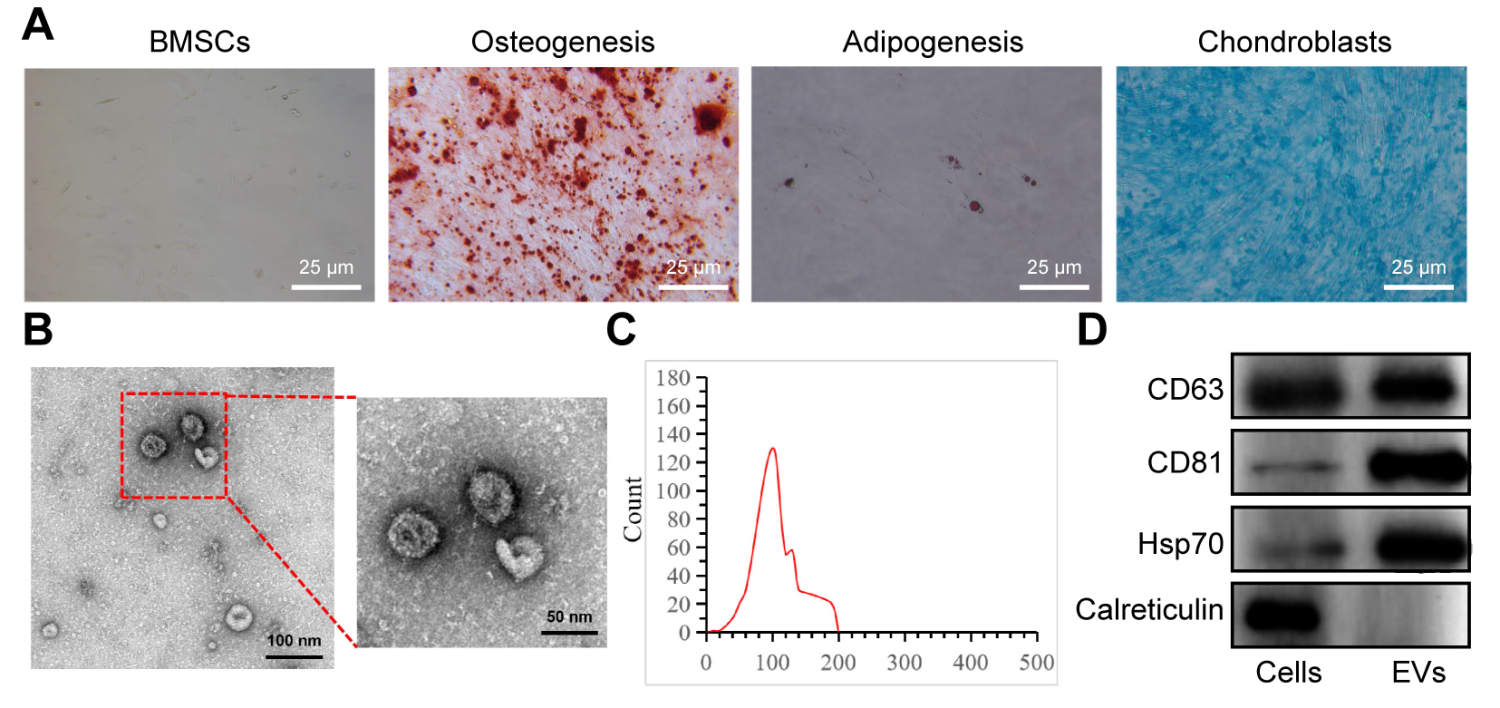
**

Supplementary Fig. 1 Identification of BMSCs and BMSCs-EVs. A, The morphological characteristics of BMSCs and their osteogenic, adipogenic, and chondrogenic differentiation observed under an optical microscope. Scale bar: 25 μm. B, The morphological characteristics of BMSCs-EVs observed under a TEM. C, Particle size of the BMSCs-EVs analyzed by NTA. D, The expression of EV surface marker proteins in the BMSCs-EVs examined by western blot analysis. The cell experiment was repeated three times independently.

**
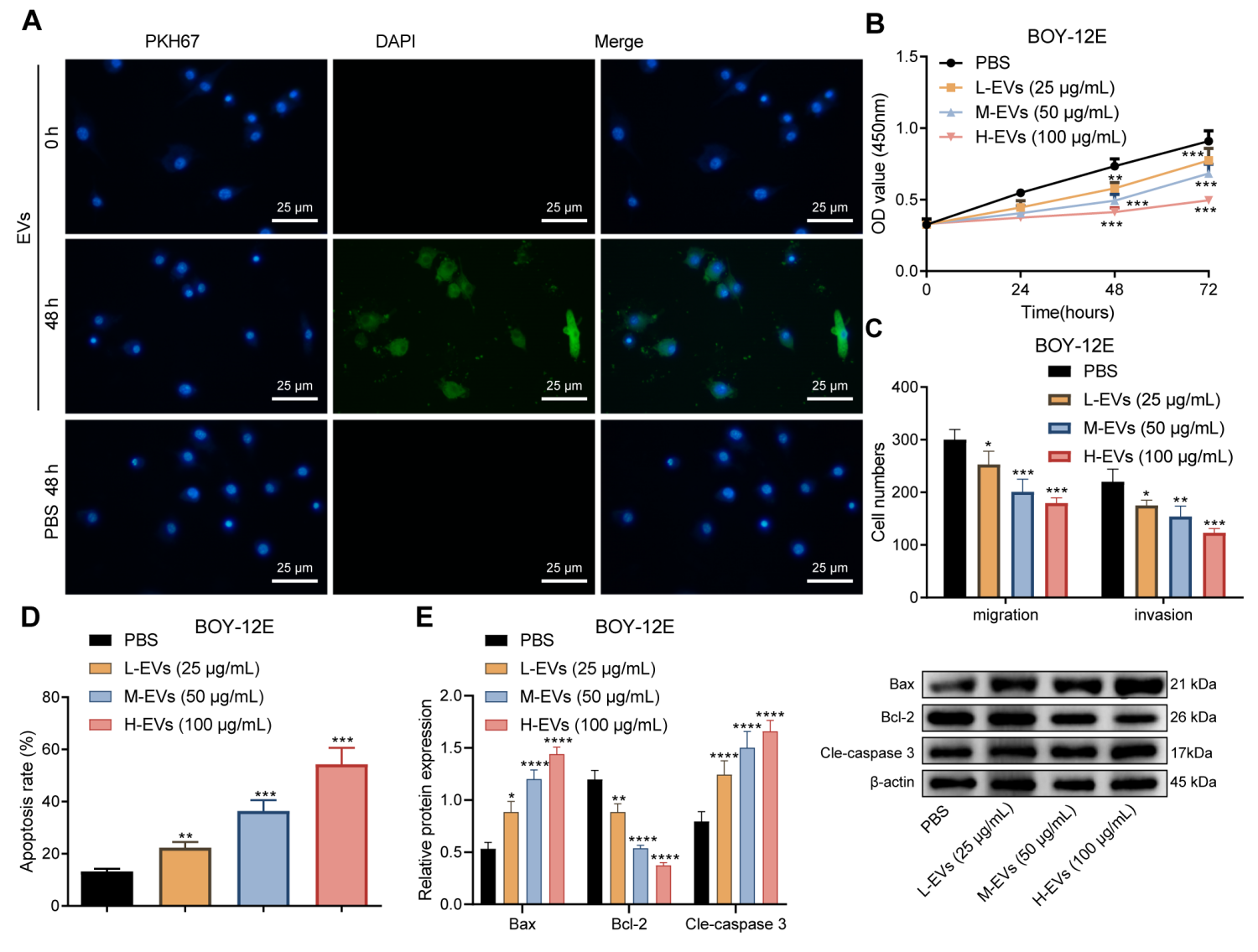
**

Supplementary Fig. 2 BMSCs-EVs inhibit the proliferation, migration and invasion of BOY-12E cells while inducing their apoptosis. A. The uptake of EVs labeled with PKH67 (green fluorescence) by the BOY-12E cells analyzed using a fluorescence microscope. DAPI stained nucleus is blue. Scale bar: 25 μm. B, The proliferation of BOY-12E cells co-cultured with different concentrations of EVs examined by CCK-8. C, The migration and invasion of BOY-12E cells co-cultured with different concentrations of EVs with BOY-12E cells tested by Transwell assay. D, The apoptosis of BOY-12E cells co-cultured with different concentrations of EVs tested by Hoechst 33342/PI staining. E, The expression of apoptosis-related factors in BOY-12E cells co-cultured with different concentrations of EVs tested by western blot analysis. Measurement data were expressed as mean ± standard deviation. Paired *t* test was conducted to compare data between the cancer tissues and adjacent normal tissues. **p* < 0.05, ***p* < 0.01, ****p* < 0.001 or *****p* < 0.0001, *vs.* PBS group. The cell experiment was repeated three times independently.

**
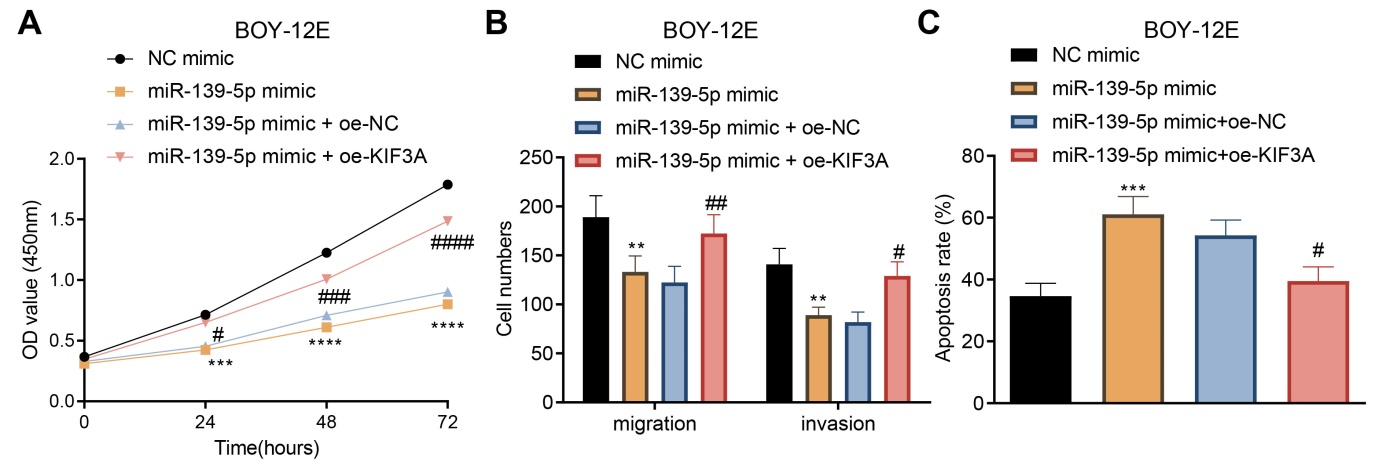
**

Supplementary Fig. 3 miR-139-5p targets KIF3A to inhibit the proliferation, migration and invasion of BOY-12E cells while inducing their apoptosis. A, The proliferation of BOY-12E cells transfected with miR-139-5p mimic or combined with oe-KIF3A examined by CCK-8. B, The migration and invasion of BOY-12E cells transfected with miR-139-5p mimic or combined with oe-KIF3A examined by Transwell assay. C, The apoptosis of BOY-12E cells transfected with miR-139-5p mimic or combined with oe-KIF3A examined by Hoechst 33342/PI staining. Measurement data were expressed as mean ± standard deviation. Unpaired *t* test was performed for two-group data comparison. One-way ANOVA was conducted for multi-group data comparison, followed by Tukey's post hoc test. **p* < 0.05, ***p* < 0.01, ****p* < 0.001 or *****p* < 0.0001, *vs.* NC mimic group. #*p* < 0.05, ^##^*p* < 0.01, ^###^*p* < 0.001, or ^####^*p* < 0.0001, *vs.* miR-139-5p mimic + oe-NC group. The cell experiment was repeated three times independently.

**
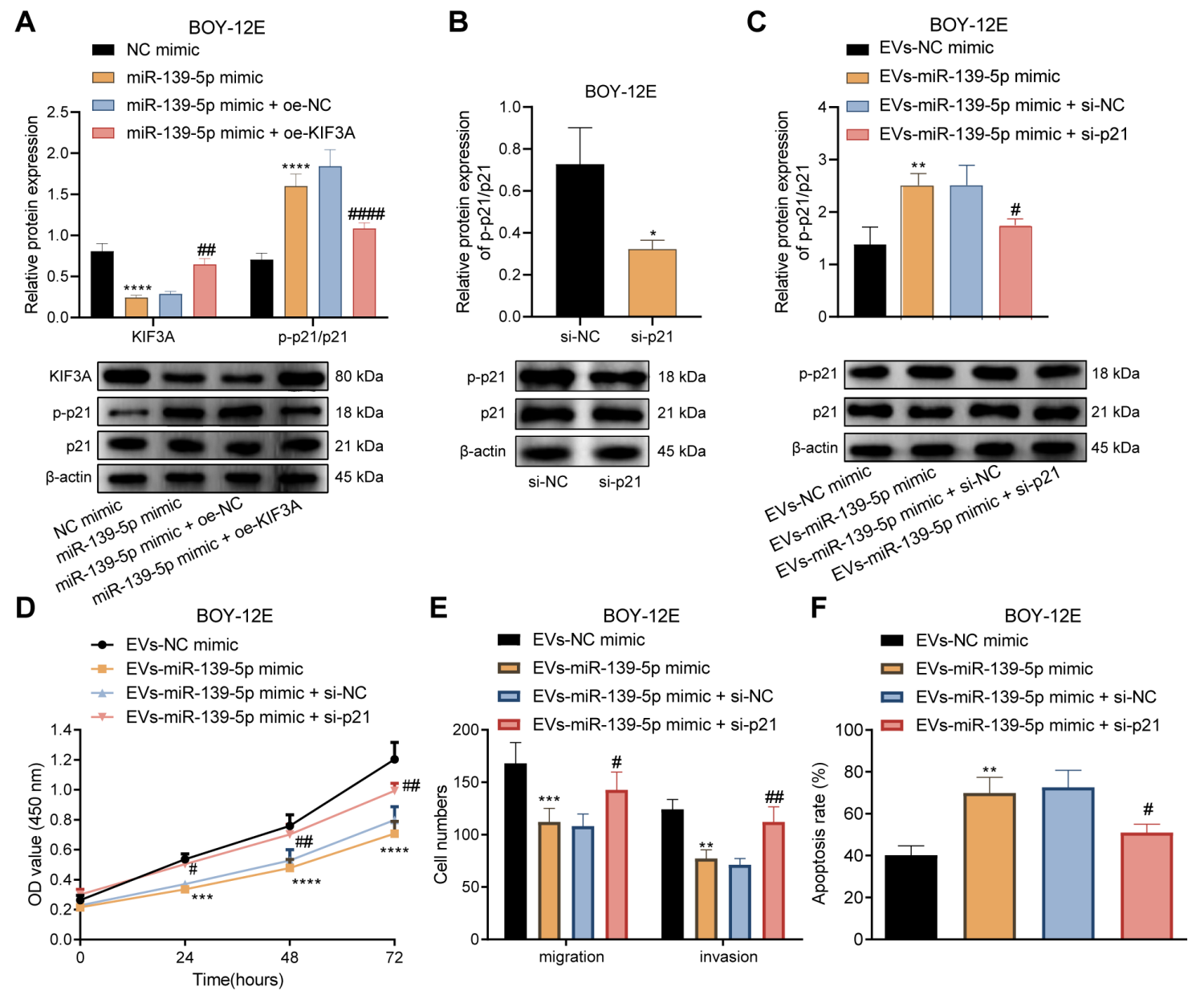
**

Supplementary Fig. 4 miR-139-5p delivered by BMSCs-EVs down-regulates KIF3A and activates p21 to inhibit the proliferation, migration and invasion of BOY-12E cells while inducing their apoptosis. A. The expression of KIF3A and the ratio of p-p21/p21 in BOY-12E cells transfected with miR-139-5p mimic or combined with oe-KIF3A detected using western blot analysis. *****p* < 0.0001, *vs.* NC mimic group. ^##^*p* < 0.01 or ^####^*p* < 0.0001, *vs.* miR-139-5p mimic + oe-NC group. B, Transfection efficiency of si-p21 in BOY-12E cells detected using western blot analysis. **p* < 0.05, *vs.* si-NC group. C, The ratio of p-p21/p21 in BOY-12E cells treated with EVs-miR-139-5p mimic or combined with si-p21 examined by western blot analysis. D, The proliferation of BOY-12E cells treated with EVs-miR-139-5p mimic or combined with si-p21 examined by CCK-8. E, The migration and invasion of BOY-12E cells treated with EVs-miR-139-5p mimic or combined with si-p21 examined by Transwell assay. F, The apoptosis of BOY-12E cells treated with EVs-miR-139-5p mimic or combined with si-p21 examined by Hoechst 33342/PI staining. Measurement data were expressed as mean ± standard deviation. Unpaired *t* test was performed for two-group data comparison. One-way ANOVA was conducted for multi-group data comparison, followed by Tukey's post hoc test. In panels C-F, ***p* < 0.01, ****p* < 0.001 or *****p* < 0.0001, *vs.* EVs-NC mimic group. ^#^*p* < 0.05 or ^##^*p* < 0.01, *vs.* EVs-miR-139-5p mimic + si-NC. The cell experiment was repeated three times independently.
